# Redox‐Reversible Iron Orthovanadate Cathode for Solid Oxide Steam Electrolyzer

**DOI:** 10.1002/advs.201500186

**Published:** 2015-12-03

**Authors:** Lizhen Gan, Lingting Ye, Cong Ruan, Shigang Chen, Kui Xie

**Affiliations:** ^1^ Key Lab of Design and Assembly of Functional NanostructureFujian Institute of Research on the Structure of MatterChinese Academy of SciencesFuzhouFujian350002P.R. China; ^2^ Fujian Provincial Key Laboratory of NanomaterialsFujian Institute of Research on the Structure of MatterChinese Academy of SciencesFuzhouFujian350002P.R. China; ^3^ School of Mechanical and Automobile Engieering and School of Materials Science and EngineeringHefei University of TechnologyHefeiAnhui230009P.R. China

**Keywords:** cathodes, FeVO_4_, FeV_2_O_4_, solid oxide electrolyzers, spinels

## Abstract

**A redox‐reversible iron orthovanadate cathode** is demonstrated for a solid oxide electrolyser with up to 100% current efficiency for steam electrolysis. The iron catalyst is grown on spinel‐type electronic conductor FeV_2_O_4_ by in situ tailoring the reversible phase change of FeVO_4_ to Fe^+^FeV_2_O_4_ in a reducing atmosphere. Promising electrode performances have been obtained for a solid oxide steam electrolyser based on this composite cathode.

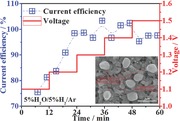

Solid oxide electrolyzers have demonstrated a tremendous advantage in the electrochemical conversion of H_2_O into H_2_ with high efficiencies by renewable electrical energy.^1^, ^2^, ^3^ The high operation temperature leads to favorable kinetics and thermodynamics. The conventional Ni–YSZ (YSZ: 8% Y_2_O_3_ stabilized ZrO_2_) cathode has exhibited excellent steam‐electrolysis performance under a reducing atmosphere; however, the Ni‐cermet is not redox stable and requires a significant concentration of reducing gas flowing over the Ni metal, avoiding the oxidation from Ni to NiO. Barnett et al. reported the high syngas yield of 7 sccm cm^−2^ with Ni–YSZ cathode from the coelectrolysis of CO_2_/H_2_O with 25% H_2_ flowing in the cathode under 1.3 V at 800 ºC.^4^ However, the Ni‐cermets do display some disadvantages, such as reduction of three‐phase boundaries (TPB) due to agglomeration after long operation and poor redox cycling causing volume instability.^5^, ^6^ The shortage of reducing atmosphere would not only breed the loss of electronic conductivity but also, in all probability, the mechanical failure of the Ni–YSZ cathode in the process of H_2_O electrolysis.^7^

Perovskite La_1–*x*_Sr*_x_*VO_3_ (LSV) has been recognized as a potential solid oxide fuel cell anode material which also has promising prospect for eventual application in solid oxide electrolyzers. As reported by Chan, Tao and Frade,^2^, ^8^, ^9^ LSV has a high electronic conductivity with typical n‐type conduction behavior in a broad temperature range of 500–1000 °C in reducing atmospheres. The sulphur tolerance of LSV has also led to a considerable improvement of electrode performance for hydrocarbon‐fueled SOFC.^10^ Very recently, Gorte et al. reported the impregnation of LSV into porous YSZ scaffold to create the electronically percolating network and remarkably outstanding fuel cell performances were obtained.^11^ However, the decomposition of LSV with the formation of insulating Sr_3_V_2_O_8_ under oxidizing atmospheres completely restricts its wide applications for fuel electrodes.^12^ To mitigate the redox instability, the stable ortho‐ and pyro‐vanadates in oxidizing atmospheres would be considered as fuel electrode candidates. However, these vanadates are not stable in a reducing environment from intermediate to high temperatures. Tao et al. reported that Ce_0.9_Ca_0.1_VO_4_ and Ce_0.8_Ca_0.2_VO_4_ were redox stable at temperatures only below 600 °C while the electrical conductivity was as low as 0.1–1 S cm^−1^ in reducing atmospheres.^13^

Spinel oxides attract a great of interest as excellent candidates for protection layer on ferric stainless steel interconnects, because of their high electrical conductivity, satisfactory thermal and structural stability.^14^, ^15^, ^16^ Spinel anode/cathode would therefore offer a reasonable structural compatibility to interconnect coatings.^17^, ^18^ Liu et al. reported that the polarization resistances of single cells based on spinel oxide Mn_2_CoO_4_, Mn_1.5_Co_1.5_O_4_ and MnCo_2_O_4_ cathodes were 1.06, 0.71 and 2.46 Ω cm^2^ at 800 ºC, respectively.^17^ Irvine et al. reported an important advance that the chromium‐rich spinel MnFeCrO_4_ was initially used as fuel electrode and this oxide demonstrated the preferable chemical stability and electrical conductivity both in reducing and oxidizing atmospheres.^19^ The chromium rich spinel (MnFeCrO_4_) can be used as an electrode support material, either alone or impregnated with conventional (La_0.75_Sr_0.25_)_0.97_Cr_0.5_Mn_0.5_O_3–δ_, La_0.8_Sr_0.2_FeO_3–δ_, Ce_0.9_Gd_0.1_O_2–δ_, CeO_2_ and/or Pd. In these initial studies, all of the impregnated spinel electrodes show a considerably enhanced performance and stability to a sufficient level for SOFC applications.

Spinel FeV_2_O_4_ with a layered structure is compatible to the spinel protection layer of ferric stainless steel interconnect. The V(3+) offers 2 free electrons to act as charge carriers for electronic conduction. In the layered structure, the V ion has a fixed coordination number of 6 in edge‐ or facet‐shared VO_6_ octahedra, which produces the V–V metal bond, overlapping with an inter‐metallic distance. This unique structure with sufficient free electrons facilitates the electron conduction in d orbitals by the edge‐ and facet‐shared octahedra. However, the spinel FeV_2_O_4_ is instable in oxidizing atmosphere at intermediate temperatures. Here, for the first time, stoichiometric amount of iron catalyst is equalized to spinel FeV_2_O_4_ to design a composite which is expected to transform to a stable single‐phase FeVO_4_ in oxidizing atmosphere at intermediate temperatures. The iron within the host lattice of FeVO_4_ is then exsolved to anchor on the surface of FeV_2_O_4_ to form catalytically active metallic Nano‐particles or Micron‐particles under reducing conditions. Upon re‐oxidation, the iron can be re‐incorporated into the host lattice, yielding a regenerative catalyst. In this case, any possible agglomeration of exsolved metallic iron nanoparticles on the substrate surface could be avoided by periodically exposing the material to oxidizing conditions.^20^

In this work, for the first time, we report a redox‐reversible iron orthovanadate cathode for solid oxide steam electrolyzer. The reversible phase changes between Fe/FeV_2_O_4_ composite and FeVO_4_ are investigated as well as the electrical and electrochemical properties of Fe/FeV_2_O_4_. High temperature steam electrolysis is then performed with this composite cathode.

**Figure** **1**a,b show the x‐ray diffraction (XRD) Rietveld refinement patterns of FeVO_4_ before and after reduction, respectively. In Figure 1a, the refinement data and the experimental results prove the pure phase of FeVO_4_ powder sample. The cell parameters of FeVO_4_ are *a* = 6.699 Å, *b* = 8.039 Å and *c* = 9.326 Å, which are well consistent with triclinic structure and space group of P‐1 (space group (SG) number of 2; powder diffraction file (PDF): 71–1592). In Figure 1b, the cell para­meters of FeV_2_O_4_ are *a* = *b* = *c* = 8.444 Å, and that is also pretty consistent with the cubic structure and space group of Fd‐3m (SG number of 227, PDF: 75–0317). A high intensity peak at 2*θ* = 44.6° corresponds to iron (PDF: 87–0721), which indicates the successful transformation from FeVO_4_ to FeV_2_O_4_ and iron after reduction. Figure 1c,d show the XRD Rietveld refinement patterns of FeVO_4_ after two redox cycles. The FeV_2_O_4_ and iron composites are repeatedly transformed to a single‐phase FeVO_4_ after heat treatment in air, while the iron catalyst again grows and anchors on FeV_2_O_4_ surface after reduction. In this work, the in situ growth of iron catalyst on FeV_2_O_4_ surface is confirmed to be completely reversible.

**Figure 1:**
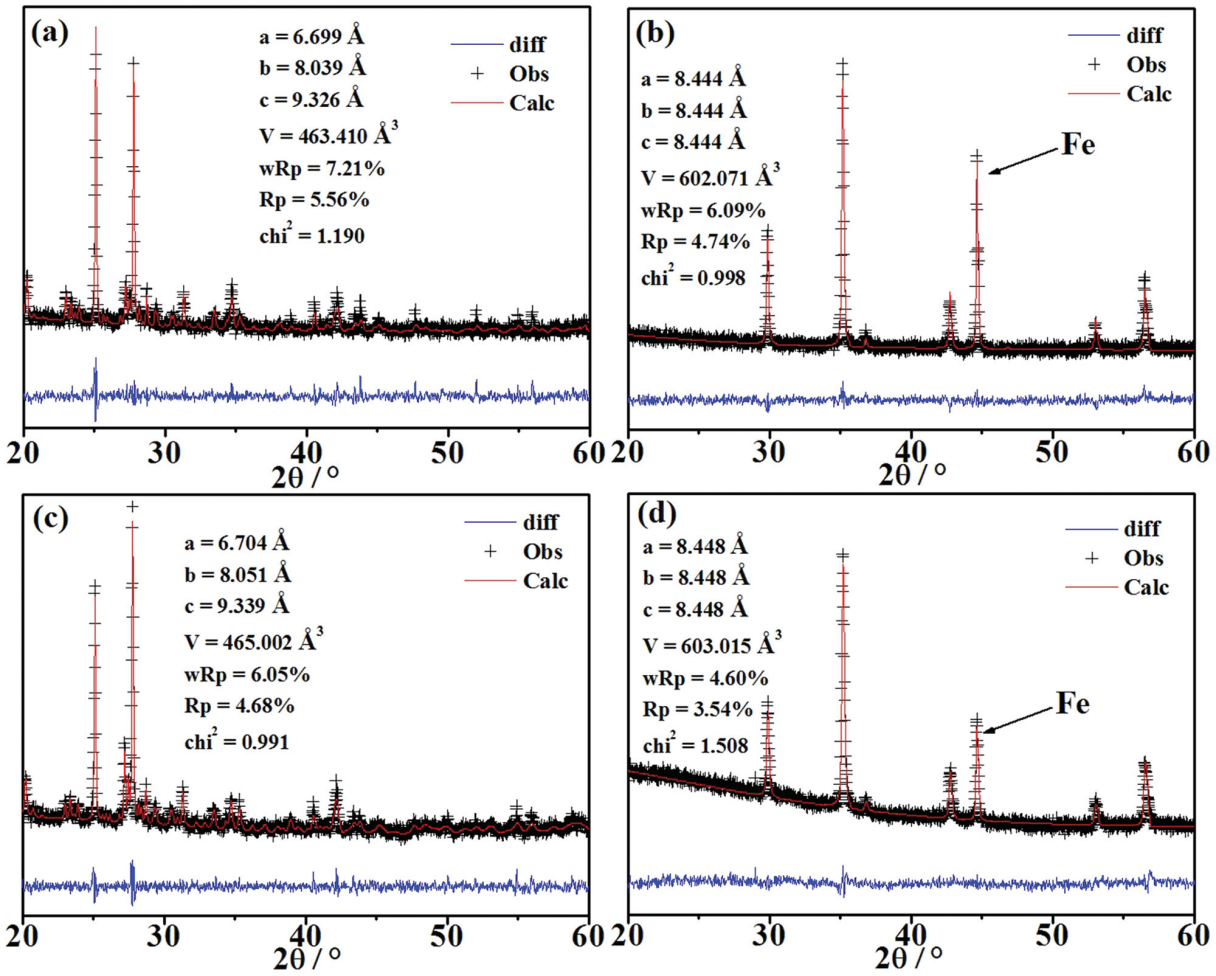
XRD Rietveld refinement patterns of a) the oxidized FeVO_4_, b) the reduced FeVO_4_ (FeV_2_O_4_ + Fe), c) the FeVO_4_, and d) the reduced FeVO_4_ (FeV_2_O_4_ + Fe), after two redox cycles.

**Figure** **2**a shows the high resolution transmission electron microscope (HR‐TEM) images of the reduced FeVO_4_ sample after two redox cycles. The lattice spacing of the substrate is measured to be 0.2519 nm, which is consistent properly with the interplanar spacing of (3 1 1) of FeV_2_O_4_. The presence of iron on FeV_2_O_4_ surface can be confirmed by the lattice spacing of 0.2015 nm (1 1 0) for iron metal (PDF: 87–0721). The HR‐TEM analysis proves the reversible in situ growth of iron catalyst on FeV_2_O_4_ surface by treating the FeVO_4_ in a reducing atmosphere. The anchored interface is expected to ameliorate the catalyst stability and optimize electrocatalytic performance.

**Figure 2:**
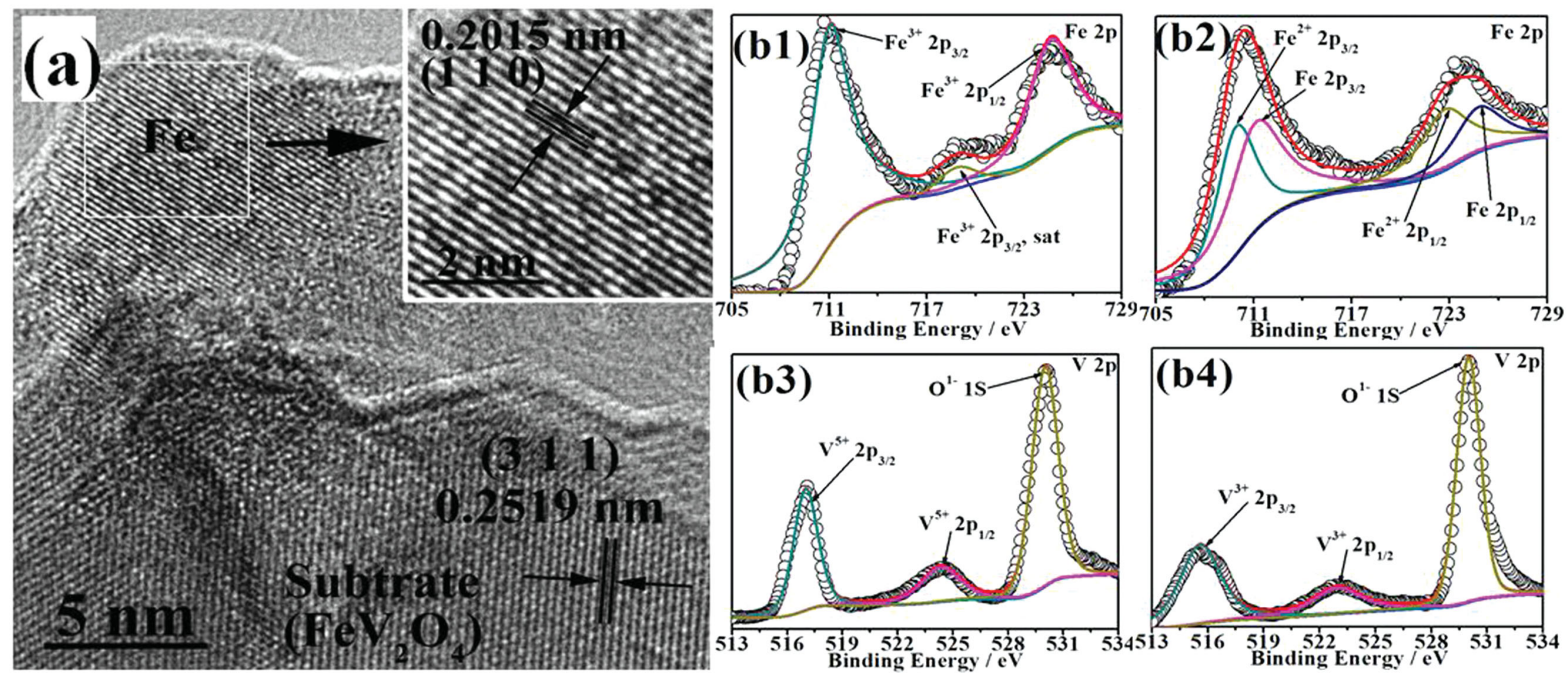
a) HR‐TEM images of the reduced FeVO_4_ (FeV_2_O_4_ + Fe). b1, b3) Fe 2p and V 2p XPS of the oxidized FeVO_4_. b2, b4) Fe 2p and V 2p XPS of the reduced FeVO_4_ (FeV_2_O_4_ + Fe).

Figure 2b1–b4 show the Fe‐2p and V‐2p XPS spectra of FeVO_4_ before and after reduction after two redox cycles. All of these XPS spectra are fitted through a Shirley‐type background subtraction method with the background functions for different spectra of the elements fitted by 80% Gaussian and 20% Lorenzian. The binding energies are calibrated to C 1s peak at 285 eV. The core level XPS spectra of Fe 2p are displayed in Figure 2b1. In Figure 2b1, three peaks at 711.0 eV, 724.6 eV and 718.8 eV are demonstrated to be Fe^3+^ 2p^3/2^, 2p^1/2^ and the satellite peak of Fe^3+^ 2p^3/2^, respectively.^21^ In Figure 2b2, it is verified that all the Fe^3+^ are transformed to Fe^2+^ and Fe^0^. The peaks for Fe^2+^ are observed at 709.9 eV for 2p^3/2^ and 722.7 eV for 2p^1/2^. In contrast, the peaks for Fe^0^ are observed at 711.1 eV for 2p3/2 and 724.8 eV for 2p^1/2^.^21^ The ratio of Fe^2+^/Fe^0^ is approximately 49/51, which further proves the complete transformation from FeVO_4_ to FeV_2_O_4_ and Fe. Figure 2b3,b4 show the core level XPS spectra of V 2p. Both spectra present a typical two‐peak structure because of the spin‐orbit splitting.^22^ In Figure 2b3, all of the vanadium of FeVO_4_ sample present the chemical state of +5, and the 2p^3/2^ and 2p^1/2^ peaks are observed at 517.0 eV and 524.3 eV (2p^3/2^ + 7.3 eV), respectively.^23^ In Figure 2b4, an obvious degeneration in binding energies for V 2p peaks is detected in contrast to the oxidized FeVO_4_ sample in Figure 2b3. The peaks at 515.6 eV and 522.9 eV correspond to V^3+^ 2p^3/^2 and 2p^1/2^ (2p^3/2^ + 7.3 eV), respectively.^23^ The chemical state change of V and Fe confirms the reversible transformation from FeVO_4_ to FeV_2_O_4_ and Fe after reduction.

To study the electrical properties of FeVO_4_ in oxidizing and reducing atmospheres, the dependence of DC conductivity on temperature is tested in air and 5%H_2_/Ar, respectively. The relative densities of the sintered FeVO_4_ samples have reached approximately 80% while the conductivities are accordingly normalized.^24^, ^25^, ^26^ **Figure** **3**a shows the Arrhenius plot of the conductivity of FeVO_4_, which illustrates a linear relationship between ln(*σT*) and 1000/*T*, displaying a typical semiconducting behavior. As shown in Figure 3a, the conductivity of oxidized FeVO_4_ sample climbs with temperature ranging from 300 to 800 ºC. The activation energy is 0.44 eV according to the Arrhenius plot and the conductivity reaches 0.4 S cm^−1^ at 800 ºC. The conduction mechanism of FeVO_4_ is therefore suggested to be pure electronic conduction above 500 K, as reported in the previous work of Gupta et al.^27^ From an atomic scale, the electronic conduction in FeVO_4_ is caused by electron hopping on equivalent iron lattice sites and Fe^2+^–O–Fe^3+^ pairs.^28^ Figure 3b shows the conductivity of reduced FeVO_4_ in 5%H_2_/Ar as a function of temperature plotted in Arrhenius form. The conductivity linearly climbs versus increasing temperature with the activation energy of 0.19 eV. The total conductivity reaches approximately 12 S cm^−1^ at 800 ºC. According to the above analysis, FeVO_4_ turns into FeV_2_O_4_ with anchored Fe particles after reduction, which implies that FeV_2_O_4_ contributes to the conductivity though iron particles are dispersively anchored on the surface of reduced sample. The conductivity of spinel FeV_2_O_4_ is approximately two orders of magnitude higher than that of MnFeCrO_4_ and LSCM ((La_0.75_Sr_0.25_)_0.95_Cr_0.5_Mn_0.5_O_3–δ_) as reported by Irvine et al. (0.4 and 0.5 S cm^−1^ in 5% H_2_/Ar at 850 ºC for MnFeCrO_4_ and LSCM, respectively).^19^, ^29^

**Figure 3:**
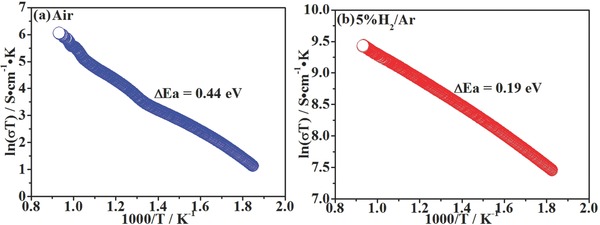
The total conductivities of FeVO_4_ versus temperature in a) air and b) 5%H_2_/Ar, respectively.

**Figure** **4** shows the field emission scanning electron microscope (FESEM) images and energy‐dispersive X‐ray spectroscopy (EDS) maps of FeVO_4_ sample before and after reduction. As shown in Figure 4a–d, the Fe and V elements are homogeneously dispersed in the oxidized sample as confirmed by EDS mapping, which indicates that no element agglomeration or phase segregation appears. Figure 4b shows many ferrous metal particles anchoring on the surface of the reduced sample spinel FeV_2_O_4_ according to EDS mapping, further indicating the in situ growth of iron catalyst on the surface of electronic conductor. The substrate still displays the homogeneously dispersion of Fe and V elements in the EDS map which is gained though the exsolution of iron particles after reduction, considered to be consistent well with the analysis stated above. In summary, the reversible in situ growth of ferrous metal particle on the spinel FeV_2_O_4_ substrate can be successfully achieved, which is expected to enhance the electrocatalytic activity of the composite after reduction.

**Figure 4:**
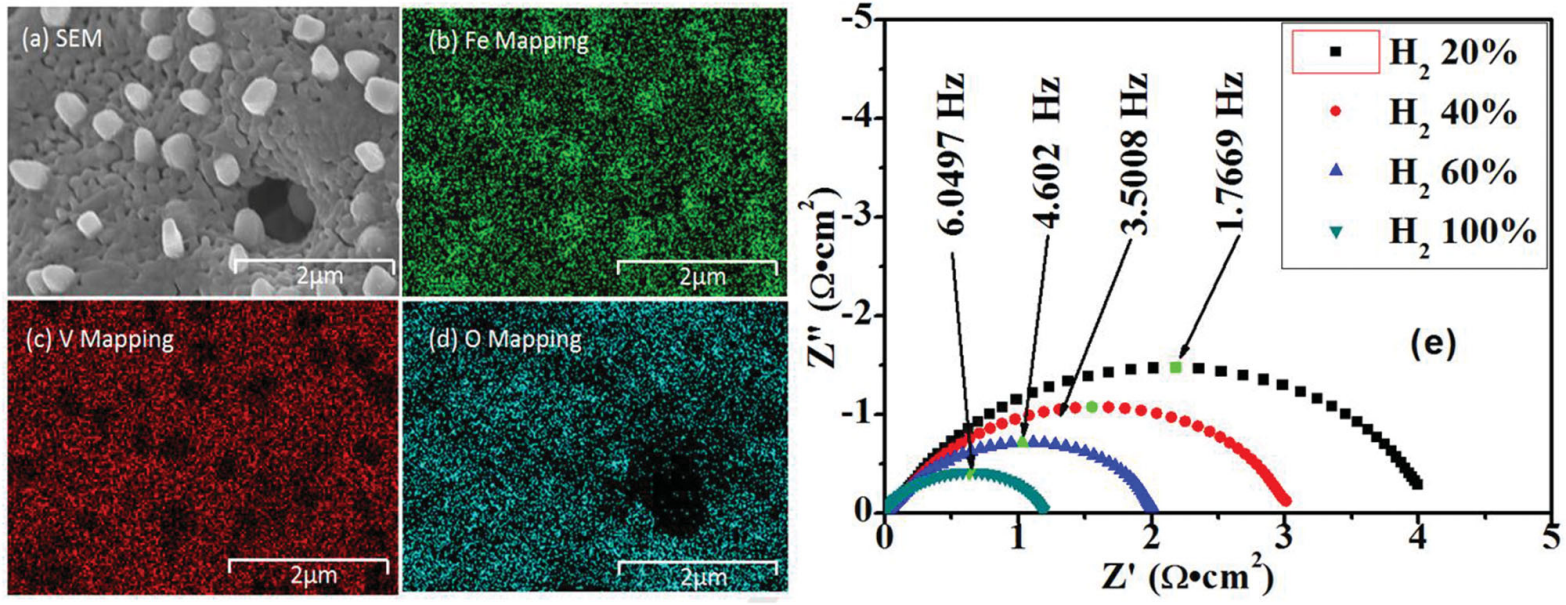
a–d) FESEM images and EDS maps of the reduced FeVO_4_. e) AC impedance spectra of symmetrical cell with FeVO_4_ under different hydrogen partial pressure at 800 ºC.

Figure 4e shows the AC impedance spectra of symmetrical cell with FeVO_4_ electrode in reducing atmospheres at 800 ºC. In this case, all the series resistances (*R*_s_) have been set as 0 to compare the polarization resistances (*R*_p_) which are calculated by Zview software.^30^ According to above analysis, the FeVO_4_ transforms to FeV_2_O_4_/Fe in the reducing atmosphere at 800 ºC. The *R*_p_ of the FeV_2_O_4_/Fe (FeVO_4_) electrode is improved from 2.0735 to 0.6005 Ω cm^2^ with *p*H_2_ rising from 20 to 100%, which indicates that the stronger reducing atmosphere effectively enhances the electrocatalytic activity of FeV_2_O_4_ and therefore minimizes the electrode polarizations. The electro‐catalytic activity of the composite electrode is comparable to LSCM and better than the chromium‐rich spinel MnFeCrO_4_ in stronger reducing atmospheres.^19^, ^29^ However, the *R*_p_ of the FeV_2_O_4_/Fe composite cathode is still lower than conventional Ni–YSZ and perhaps the limited catalytic activity and insufficient porosity can account for this phenomenon. The *R*_p_ for Ni–YSZ is around 0.1–0.5 Ω cm^2^ under reducing conditions, though the Ni–YSZ can be oxidized by steam more easily in reducing atmospheres.^31^, ^32^, ^33^, ^34^, ^35^

**Figure** **5** shows the typical curves of current density versus voltage (*I–V* curves) of the electrolyzers based on FeVO_4_ cathode for steam electrolysis with 5%H_2_O/5%H_2_/Ar and 5%H_2_O/Ar fed to cathodes, respectively, at 800 ºC. The open circuit voltages (OCVs) are 0.92 V in 5%H_2_O/5%H_2_/Ar and 0.37 V in 5%H_2_O/Ar, which are dramatically consistent with the open circuit voltages for oxygen concentration cells.^36^ The high OCVs show the good separation between the anodic and cathodic gases. As shown in Figure 5a, the current density reaches 0.12 A cm^−2^ under 1.5 V in 5%H_2_O/5%H_2_/Ar, which is approximately 100% higher than the reported values for steam electrolysis with LSCM cathode in our previous works,^36^, ^37^ further verifying the advantage of electrocatalytic activity of composite Fe/FeV_2_O_4_ cathode. As exhibited in Figure 5a, direct steam electrolysis with 5%H_2_O/Ar begins at 1.0 V and the current density finally reaches 0.10 A cm^−2^ under 1.5 V at 800 ºC, which is still 80% higher than the performance of LSCM cathode (0.043 A cm^−2^ under 1.5 V at 800 ºC in 3%H_2_O/Ar.^38^ The direct steam electrolysis has been achieved with this composite cathode though the performance is still lower than those of reported researches concerning those traditional Ni–YSZ cathodes. The current densities have reached approximately 1.0–1.5 A cm^−2^ for solid oxide electrolyzers with thin‐membrane YSZ electrolytes and under 1.5 V at 800 ºC.^31^, ^32^, ^33^, ^34^, ^35^ The high performance is mainly attributed to the lower ionic resistance of thin YSZ electrolytes and the high activity of Ni–YSZ cathodes. However, these Ni–YSZ cathodes can be oxidized in reducing atmospheres more easily. In this work, the synergetic effect of catalytic active iron particle and reducing‐stable FeV_2_O_4_ contributes to the improved performances in contrast to LSCM cathodes. It is noteworthy that significant change in slope is observed at approximately 0.95 V, which is therefore reasonably speculated as the onset potential of H_2_ generation through steam electrolysis. There exist two different main cell processes in the two voltage regions: (1) electrochemical reduction of cathodes and oxidation of anodes under low voltages; (2) steam electrolysis under high voltages.

**Figure 5:**
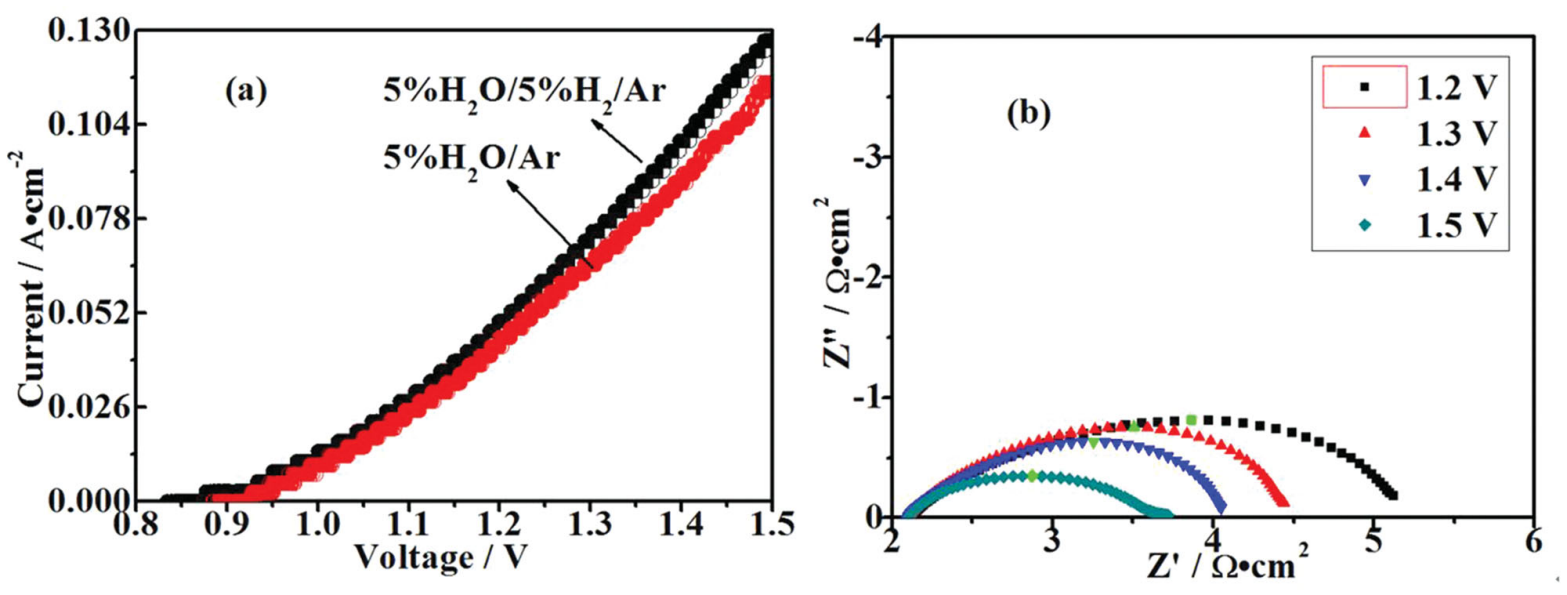
a) Current–voltage curves of the solid oxide electrolyzers with cathodes fed by 5%H_2_O/5%H_2_/Ar and 5%H_2_O/Ar, respectively, at 800 ºC. b) AC impedance spectra of the solid oxide electrolyzers with 5%H_2_O/Ar in FeVO_4_ cathode under different applied voltages at 800 ºC.

To investigate the trend of electrolyzer resistances with the applied voltages, in situ AC impedance spectra of the solid oxide electrolyzers are therefore measured at 800 ºC. As shown in Figure 5b, the *R*_s_ is 2.24 Ω cm^2^ and consistent with the ionic resistance of the YSZ electrolyte at 800 °C, which is generally stable under the whole range of voltages. However, the *R*_p_ is obviously ameliorated with the increasing voltages from 3.0679 Ω cm^2^ under 1.2 V to 1.4987 Ω cm^2^ under 1.5 V by Zview software, which indicates that the increasingly climbing voltages significantly enhance electrode activation. The *R*_s_ is principally consistent with the ionic resistance of the YSZ disk and remains stable both in strong and weak reducing atmospheres; however, the *R*_p_ in 5%H_2_O/5%H_2_/Ar is slightly smaller than that in 5%H_2_O/Ar, which is likely because of the stronger reducing atmosphere that affects electrode activity. These values regarding electrode polarization performance are around 50% to the reported values with Ni–YSZ cathode for steam electrolysis. In one reported work, the resistance is 1.126 Ω cm^2^ under similar conditions in flowing H_2_/Ar.^39^ However, the electrode resistance can be reaching above 1000 Ω cm^2^ if H_2_ is absent because of the oxidation from Ni to NiO.^7^

**Figure** **6** shows the steam electrolysis performances with current densities and H_2_ production recorded *versus* time under a series of applied voltages at 800 ºC. In Figure 6a,b, the current density plateau increases with external applied potentials from 1.1 to 1.5 V with/without a flow of reducing gas, which indicates the superior stability of the composite FeV_2_O_4_/Fe cathode both in stronger and less reducing atmospheres. The productions of H_2_ with the same increase tendency reach the maximum value of 0.7 ml min^−1^ cm^−2^ in 5%H_2_O/5%H_2_/Ar and 0.6 ml min^−1^ cm^−2^ in 5%H_2_O/Ar under 1.5 V, respectively, which are two times higher than the reported values with LSCM cathodes at 800 ºC under 1.5 V.^36^ As shown in Figure 6c,d, the current efficiency reach 98% with 5%H_2_O/5%H_2_/Ar and 92% with 5%H_2_O/Ar under 1.5 V, which is around 25–30% higher than the current efficiency of 61% with LSCM cathode in our previous work under the same condition.^36^ The loss of current efficiency for LSCM is related to the transport of impurities in YSZ electrolyte. The limited performance of LSCM electrode also facilitates the impurities transport. And the current efficiencies with this new composite Fe/FeV_2_O_4_ cathode are comparable to those of the state‐of‐art Ni–YSZ cathodes under similar conditions.^40^, ^41^, ^42^, ^43^

**Figure 6:**
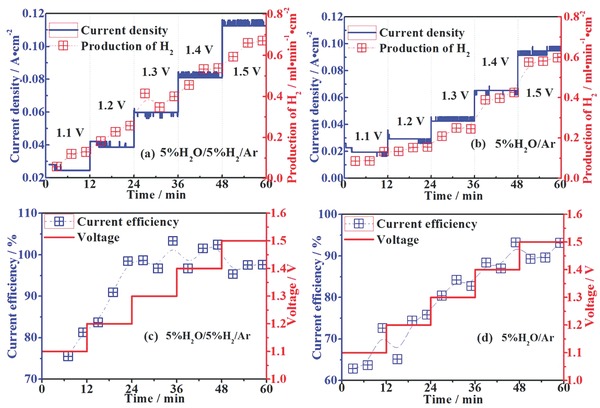
Short‐term current densities, productions of H_2_ and current efficiencies for steam electrolysis with cathodes fed by a, c) 5%H_2_O/5%H_2_/Ar and b,d) 5%H_2_O/Ar at 800 ºC, respectively.

In this work, we for the first time report a redox‐reversible iron orthovanadate FeVO_4_ cathode for solid oxide steam electrolyzer. The FeVO_4_ cathode with oxidation stability is reversibly trasformed to a reducing‐stable FeV_2_O_4_ electron conductor with iron catalyst anchoring on substrate surface upon reduction. The in situ growth of iron catalyst on highly conducting spinel oxide FeV_2_O_4_ has a possibility to shed a light on the the development of new ceramic redox‐reversible cathode. Promising electrode performance has been demonstrated with Fe/FeV_2_O_4_ compostie. Steam electrolysis with a current efficiency as high as 98% is achieved at 800 °C. This new spinel material would provide a new pathway for redox‐reversible orthovanadate cathodes for solid oxide steam electrolyzers.

## Experimental Section

The FeVO_4_ powders were synthesized through a combustion method with stoichiometric amounts of ferric nitrate (Fe(NO_3_)_3_·9H_2_O), metavanadate (NH_4_VO_3_) and glycine to keep a molar ratio of Fe:V = 1:1, and followed by a heat treatment at 650 ºC (2 ºC min^−1^) for 6 h in air.^44^, ^45^ The phase formations were confirmed by using X‐ray diffraction (XRD, Cu K_α_, 2*θ* = 3° min^−1^, D/MAX2500V, Rigaku Corporation, Japan) and the data were refined by using the General Structure Analysis System (GSAS) software.^46^ High‐resolution transmission electron microscopy (HRTEM, JEM‐2100F, JEOL Ltd, Japan) was employed to investigate the microstructures of the samples. X‐ray photoelectron spectroscopy (XPS, ESCALAB25, Thermo, USA) with monochromatized Al Kα at *hv* = 1486.6 eV were utilized to analyze the element states. The electrical properties were examined by the DC four‐terminal method.^47^

The symmetric cell with 1‐mm‐thick YSZ electrolyte and FeVO_4_‐SDC (SCD: Sm_0.2_Ce_0.8_O_2_) electrode was made by printing the composite electrode slurry onto two surfaces of the YSZ electrolyte support with an area of approximately 1 cm^2^ followed by a heat treatment at 600 ºC (2 ºC min^−1^) for 3 h in air.^48^, ^49^ The single electrolyzer with FeVO_4_‐SDC cathode and LSM‐SDC (LSM: La_0.8_Sr_0.2_MnO_3–δ_) anode was made by the same method. The cell microstructure was investigated with scanning electron microscope (SEM, JSM‐6490LV, JEOL Ltd, Japan). Electrochemical measurement of the cells was performed using an electrochemical station (IM6, Zahner, Germany) with a frequency range of 10^6^–0.1 Hz and a signal strength of 10 mA. The gas flow was controlled with the mass flow meters (D08–3F, Sevenstar, China). The steam electrolysis test was performed by sealing the electrolyzer in a home‐made testing jig. Electrochemical measurements including AC impedance (10^6^–0.1 Hz, 10 mV) and current–voltage (*I*–*V*, 0.006 V s^−1^) curves were tested (two‐electrode type) with cathodes fed by 5%H_2_O/5%H_2_/Ar or 5%H_2_O/Ar (15 ml min^−1^) at 800 ºC. The hydrogen production was analyzed by using an online gas chromatograph (GC2014, Shimazu, Japan).
